# Comparative levels of salivary oxidative stress markers in oral precancer and oral cancer patients

**DOI:** 10.6026/973206300214962

**Published:** 2025-12-15

**Authors:** Rashmi Christin Kerketta, Pradeep Kumar Yadav, Litha T, Maheshwari Shanmugam, Susmit Sneha, Kohinoor Acharya, Miral Mehta

**Affiliations:** 1 Department of Oral Pathology & Microbiology, Government Dental College, Raipur, Chhattisgarh, India; 2 Dental officer, ECHS Polyclinic, Jashpur, Chhattisgarh, India; 3 Department of Oral Pathology and Microbiology, Farooqia Dental College & Hospital, Mysuru, Karnataka, India; 4 Department of Oral Pathology and Microbiology, JKKN Dental College and Hospital, Komarapalayam, India; 5 Medical Officer (NOHP), Uttar Pradesh Government, Uttar Pradesh, India; 6 Department Oral Maxillofacial Surgery, HI-Tech Dental College and hospital, Bhubaneswar, Odisha, India; 7 Department of Pediatric and Preventive Dentistry, Karnavati School of Dentistry, Karnavati University, Gandhinagar, Gujarat, India

**Keywords:** Saliva, stress markers, oral pre cancer, cancer

## Abstract

Oxidative stress is a major factor in oral carcinogenesis, but a comparative comparison of salivary biomarkers in different stages of disease development has not been fully described. This case-control study compared the levels of salivary oxidative stress in 180 participants (n= 60 healthy controls, n=60 oral precancer and n=60 oral cancer). The malondialdehyde (MDA), superoxide dismutase (SOD), catalase (CAT), glutathione peroxidase (GPx) and total antioxidant capacity (TAC) in saliva were measured by spectrophotometric and enzyme assays. The outcome showed greatly high levels of MDA and exhausted antioxidant enzyme activities in patients with cancer in comparison to the precancer and control conditions (p<0.001) and there was progressive worsening in relation to the progress of disease. Thus, we show the possible application of salivary oxidative stress measurements to be used as a non-invasive diagnostic and prognostic method of oral cancer to aid in the early diagnosis and risk stratification of susceptible groups.

## Background:

Oral cancer is the sixth most prevalent malignancy in the world with about 377,000 new cases being reported every year and the burden causing high morbidity and mortality especially in the developing countries [1]. With the improvement in diagnostic and therapeutic treatment, the five year survival rate of oral squamous cell carcinoma (OSCC) is about 50-60, which is mostly due to late diagnosis and presentation of the disease [2]. The oral carcinogenesis process is usually a multistep process that passes through clinically definitive precancerous lesions such as oral leukoplakia, erythroplakia and oral submucous fibrosis (OSMF), which has critical intervention windows to block malignant progression [3]. An imbalance between reactive oxygen species (ROS) production and antioxidant defense mechanisms, known as oxidative stress, has been considered as one of the core causes of carcinogenesis [4]. Overproduction of ROS causes lipid peroxidation, oxidation of proteins as well as DNA damage that may induce and enhance neoplastic transformation [5]. Oxidative stress is prone to the oral cavity because of constant exposure to exogenous carcinogens such as tobacco, alcohol and betel quid that significantly increases the rate of ROS production and reduces the levels of antioxidants [6]. Saliva has become a popular diagnostic system with many advantages over serum: non-invasive collection, minimal patient discomfort, economical and presence of biomarkers indicative of local and systemic diseasing conditions [7]. Recent studies have shown that the composition of the salivary reflects pathological changes in oral tissues and thus a perfect tool of biomarker discovery in oral disease [8]. Various pieces of literature have investigated oxidative stress indicators in saliva of the oral cancer patients separately, with the results showing increased malondialdehyde (MDA) levels and decreased activities of antioxidant enzymes [9, 10]. The steady end-product of lipid peroxidation is malondialdehyde which is an effective indicator of oxidative cell damage [11]. The major enzymatic defense mechanism of oxidative damage consists of antioxidant enzymes such as superoxide dismutase (SOD), catalase (CAT) and glutathione peroxidase (GPx) enzymes [12]. Cases of this fine balance between pro-oxidants and antioxidants disruption has been attributed to oral carcinogenesis, but the changes that occur at different stages of the disease have not been fully explained. Past studies by Shamsi *et al.* illustrated that salivary levels of MDA were much higher in OSCC patients than in the healthy controls [13]. In the same vein, Choudhari *et al.* have reported reduced levels of antioxidant enzyme activities in oral cancer patients which imply the impairment of antioxidant defence mechanisms [14]. Nonetheless, the majority of the available literature has been on binary comparisons between cancer patients with healthy controls, but not much work has looked at the spectrum of oxidative stress modifications between normal mucosa and precancerous conditions to the pathogenic malignancy. All the information about the patterns of oxidative stress markers at the extremes of the oral disease progression may yield useful information on the carcinogenesis mechanism and may also present possible biomarkers of early disease and risk classification. Moreover, simultaneous evaluation of several parameters of oxidative stress could be more diagnostic and prognostic than individual evaluation using a single marker [15]. Although salivary diagnostics has recently gained increased interest, few studies have been able to combine various oxidative stress indicators in different disease-progression stages into one study, using consistent methodology and sufficient sample sizes. Therefore, it is of interest to comparatively measure the salivary oxidative stress markers, such as malondialdehyde, superoxide dismutase, catalase, glutathione peroxidase and total antioxidant capacity in healthy controls, patients in the oral precancer stage and patients with oral cancer.

## Materials and Methods:

The statistical power analysis was used to estimate the size of the sample with the error of 0.05, a power of 90, the expected effect size of 0.40 using previous research that had analyzed salivary oxidative stress considerations. The estimated minimum sample size was 51 subjects in each group. There was a 60 sample per group to ensure consideration of the possible sample degradation and technical losses and it was estimated that a total of 180 subjects would take part in the study.

## Groupings and recruitment of participants:

## The participants were divided into three categories:

Group I (Healthy Controls, n=60): Healthy age and gender-matched people with no clinical signs of any oral mucosal lesions, no systemic illnesses and no tobacco/alcohol history.

Group II (Oral Precancer, n=60): The patients were found to have oral potentially malignant disorders, namely leukoplakia (n=28), oral submucous fibrosis (n=22) and erythroplakia (n=10), which were verified by clinical examination and histopathological evidence of epithelial dysplasia.

Group III (Oral Cancer, n=60): Treatment naive patients with the histopathological diagnosis of oral squamous cell carcinoma who have been diagnosed within the past four weeks.

## Inclusion criteria:

In the case of Groups II and III: age between 25-70 years; a diagnosis of the condition is proven by histopathology; no previous treatment of the condition; there is sufficient saliva production; the patient is willing to take part. In case of Group I: 25-70 years old; the oral mucosa is clinically healthy; there is no history of systemic diseases; no bad oral habits.

## Exclusion criteria:

All groups: systemic diseases, such as diabetes mellitus, cardiovascular disorders, autoimmune disorders, or chronic inflammatory disorders; current intake of antioxidant supplements, vitamins, or anti-inflammatory drugs; pregnancy or lactation; active periodontal disease; dental procedures within four weeks before the collection of saliva; radiation directed to the head and neck area; intake of food or beverages in the 2-hour period before saliva collection.

## Saliva collection protocol:

Whole saliva samples were not stimulated and the sample was collected at 9:00 to 11:00 AM to reduce circadian differences. The respondents were told to avoid eating, drinking, smoking or oral hygiene activities at least two hours before their collection. Respondents were asked to sit in an upright position and tell them to wait one minute and saliva to amass on the floor of the mouth and then drool into sterile graduated collection tubes. Saliva that had been collected was about 5 mL in volume that was obtained in 10-15 minutes. Samples were put on ice and taken to the laboratory within 30 minutes.

## Sample processing/storage:

The saliva samples were centrifuged at a rate of 3000rpm over a period of 15 minutes at 4 C to eliminate cellular debris and particulates. The supernatant was obtained in the clear tube and then aliquoted into the sterile cryovials and kept at -80 C awaiting biochemical analysis. All samples were examined within a period of four weeks after collection in order to reduce the degradation.

## Biochemical analysis:

Malondialdehyde (MDA): Determined by use of thiobarbituric acid reactive substances (TBARS) method. Thiobarbituric acid was added to the samples and the absorbance at 532 nm was recorded in a spectrophotometer at 95°C. The outcomes were in nmol/mL.

## Superoxide Dismutase (SOD):

The assay was determined by the pyrogallol autoxidation inhibition. The activity of enzymes was studied based on the inhibition of pyrogallol autoxidation at 420 nm. The results were in U/mL.

## Catalase (CAT):

Measured through the breakdown of hydrogen peroxide at 240 nm. The rate of H_2_ O_2_ degradation was used to determine the enzyme activity and was expressed as U/mL.

## Glutathione Peroxidase (GPx):

Ascertained by a coupled enzyme assay system utilizing cumene hydroperoxide as a substrate. The activity of the enzymes was measured at 340 nm in units per mL.

## Total Antioxidant Capacity (TAC):

Determined by the ferric reducing ability of plasma (FRAP) salivaric adapted assay. At 593 nm, the absorbance was taken and results were converted in mmol/L equivalents.

All biochemical tests were done in duplicates and a single trained laboratory technician who was not informed of the group assignment conducted the tests. The control of quality was done using standard reference samples per batch.

## Statistical analysis:

The analysis of data was performed using the SPSS version 26.0. The descriptive statistics were presented in means, standard deviations, frequencies and percentages. Shapiro Wilk test was used to test the normality of distribution. Continuous data between the three groups were compared with one-way ANOVA and then post-hoc, Tukey honestly significant difference (HSD) test was performed to compare them in pairs. To determine connections between oxidative stress signs and clinical variables, Pearson correlation coefficient was computed. The receiver operating characteristic (ROC) curve was conducted to assess the diagnostic accuracy of single markers. All analyses were deemed as being significant with a p-value of less than 0.05.

## Results:

The study comprised 180 participants divided equally into three groups of 60 subjects each. Table 1 (see PDF) presents the demographic and clinical characteristics of study participants. The mean age was 48.3 ± 11.2 years in controls, 51.6 ± 9.8 years in precancer patients and 54.2 ± 10.4 years in cancer patients, with no significant difference among groups (p=0.089). Male predominance was observed across all groups, particularly in Groups II and III, reflecting the established gender predilection for oral malignancies. Table 2 (see PDF) demonstrates the comparative analysis of salivary oxidative stress markers among the three study groups. Mean salivary MDA levels were significantly elevated in the cancer group (6.84 ± 1.52 nmol/mL) compared to precancer (4.26 ± 1.18 nmol/mL) and control groups (2.14 ± 0.68 nmol/mL), with statistically significant differences among all pairwise comparisons (p<0.001). A progressive increase in MDA levels was evident across disease stages, indicating escalating lipid peroxidation with malignant progression. Antioxidant enzyme activities demonstrated inverse patterns, with progressive reduction from controls through precancer to cancer groups. Mean SOD activity was 8.42 ± 1.24 U/mL in controls, 5.68 ± 1.36 U/mL in precancer patients and 3.28 ± 1.08 U/mL in cancer patients (p<0.001). Similarly, catalase activity showed significant stepwise reduction: controls (12.86 ± 2.14 U/mL), precancer (8.94 ± 1.88 U/mL) and cancer (5.42 ± 1.64 U/mL) (p<0.001). Glutathione peroxidase and total antioxidant capacity exhibited comparable declining trends across disease progression stages. Subgroup analysis within the oral cancer group revealed correlations between oxidative stress markers and clinical parameters (Table 3 - see PDF). Advanced TNM staging (III-IV) was associated with significantly higher MDA levels (7.32 ± 1.48 nmol/mL) compared to early stages I-II (5.86 ± 1.24 nmol/mL) (p=0.002). Conversely, antioxidant enzyme activities were significantly lower in advanced stages. Poorly differentiated tumors demonstrated higher MDA levels (7.64 ± 1.38 nmol/mL) and lower antioxidant capacities compared to well-differentiated tumors (6.18 ± 1.42 nmol/mL) (p=0.008). Pearson correlation analysis revealed strong inverse correlation between MDA levels and antioxidant enzyme activities (r ranging from -0.684 to -0.742, p<0.001 for all comparisons). ROC curve analysis for discriminating cancer from non-cancer groups demonstrated that MDA exhibited the highest area under curve (AUC = 0.942, 95% CI: 0.906-0.978), followed by SOD (AUC = 0.916) and TAC (AUC = 0.898). A combination of MDA and SOD yielded improved discriminatory capacity (AUC = 0.968).

## Discussion:

The current work critically assessed the salivary oxidative stress indicators in the range of disease development, showing a high level of changes in pro-oxidant and antioxidant parameters, which are correlated with the severity of the disease. The results show a linear degradation in oxidative homeostasis of healthy controls through precancerous lesions to invasive malignancy which reinforces the importance of oxidative stress in oral carcinogenesis. Substantially high levels of salivary MDA among the patients with oral cancer support the results of earlier studies. Similar levels of salivary MDA were found to be high in OSCC patients according to Srivastava *et al.* which they explained by the higher level of lipid peroxidation induced by the overproduction of ROS [9]. Malondialdehyde, a stable aldehyde product of the peroxidation of the polyunsaturated fatty acids, is a dependable biomarker of the oxidative cellular damage. The MDA levels increasing continuously in the course of the disease stages witnessed in our research study is an indication of cumulative oxidative damage throughout the process of malignant transformation, which is in line with the free radical theory of carcinogenesis [16]. The marked decrease in antioxidant enzyme activities (SOD, CAT, GPx) in cancer patients over that in controls is congruent with what other studies have shown with regard to impair antioxidant defence processes of oral malignancy. Choudhari *et al.* also stated reduced SOD and catalase activities in oral cancer patients and suggested that excessive ROS generation surpasses the capability of antioxidants and caused the depletion of enzymes and oxidative cell damage [14]. Superoxide dismutase helps in the dismutation of superoxide radical to hydrogen peroxide which is later broken down by catalase to produce water and oxygen which makes up the major enzyme defense against ROS. The sequential depletion of these enzymes among our cancer patients suggests systemic depletion of antioxidants. The intermediate oxidative stress profile that was found in precancer patients is a particularly important discovery with some clinical consequences. This population had elevated levels of MDA and antioxidant activities intermediate between controls and cancer patients, thereby indicating that changes in oxidative stress are antecedents to the development of overt malignancy and could be the cause of malignant transformation. The same claim was made by Zhou *et al.* who found that a change in oxidative stress was evident in oral leukoplakia patients and that the change was more evident in dysplastic lesions [17]. These findings confirm the idea of gradual accumulation of oxidative damage in the course of multistep carcinogenesis and indicate that these markers may have a role to play in risk stratification and in keeping track of the possibility of malignant transformation.

The correlations that we have found between the markers of oxidative stress and clinicopathological parameters give us more information about the disease biology. The increase in the levels of MDA and the decrease in antioxidant capacities were connected with the further development of TNM stage and the reduction of histological differentiation, which can be proposed to indicate that the level of oxidative stress increases with tumor progression and aggressiveness. This finding is consistent with the works of Bani-Ahmed *et al.* who revealed that oxidative stress indicators correlated with tumor stage in head and neck cancer [18]. Such associations could be indicative of a high metabolic rate and production of ROS in poorly differentiated rapid proliferating tumors. Oxidative stress has complex pathophysiological mechanisms in oral cancer. Prolong use of tobacco and alcohol which are among the primary risk factors of oral cancer leads to the production of large amounts of ROS via various mechanisms such as direct oxidant provision, inflammatory cell signaling and the inability of antioxidants to metabolize [6]. Oxidative stress must have been caused by the high rate of tobacco and alcohol consumption within our cancer and precancer groups. Also, the cancer cells express disturbed metabolism that is highly glycolytic and dysfunctioning of the mitochondria producing excessive metabolic byproducts that are ROS. The oxidative stress on lipids, proteins and DNA resulting can stimulate the genetic instability, turn oncogenic signalling pathways and repress apoptosis, promoting carcinogenesis [5]. Saliva is a good place of oxidative stress biomarker measurement in oral diseases because of its direct contact with oral tissues and non-invasive availability. We discovered that salivary MDA and SOD had excellent discriminatory ability to differentiate cancer and non-cancer groups (AUC> 0.90) which can be used to diagnose cancer. The diagnostic accuracy of the combination of the multiple markers was also greater, which is in line with the idea that the use of multiparameter analysis can enhance the performance of biomarkers. These results facilitate the establishment of salivary panel of oxidative stress markers as an adjunctive diagnostic panel to the screening and early diagnosis of oral cancer, especially among high- risk groups [7]. These findings have significant clinical implications. To begin with, the evidence of an oxidative stress change in precancerous lesions represents a potential of chemoprevention interventions using oxidative pathway to prevent progression into malignant lesions. Trials of antioxidant supplementation in the high-risk populations should be investigated. Second, salivary markers of oxidative stress might be considered as non-invasive devices of measuring treatment compliance and recurrence. Third, patients with oral potentially malignant disorders with high-oxidative stress profiles might need more attention in terms of malignant transformation. Limitations of the study are the cross-sectional nature of the study, which does not allow evaluating temporal changes and causality. Mechanistic information would be useful in longitudinal research on the development of oxidative stress markers in the course of malignant transformation. Also, although we did not include patients with systemic diseases and those taking drugs that could affect oxidative states; residual confounding of diet factors and environmental exposures cannot be completely eliminated. The research sample was selected based on one tertiary care institution, which may not be widely applicable. Evidence would be enhanced with future multicenter studies with larger sample sizes and different populations. Moreover, we have measured a small set of oxidative stress indicators; the new biomarkers such as 8-hydroxy-2-deoxyguanosine, protein carbonyls and individual antioxidant factors are subject to study.

## Conclusion:

A gradual decline in salivary oxidative homeostasis in the oral disease spectrum with profound increases in lipid peroxidation and antioxidant defense depletion in cancer patients with intermediate changes throughout precancer patients and links with tumor aggressiveness is shown. These results confirm the use of salivary oxidative stress markers as a potential diagnostic and prognostic instrument to use in oral malignancies non-invasively, which can simplify the process of early disease diagnosis, risk stratification and therapeutic monitoring of oral cancers in clinical practice.
